# Potential epigenetic molecular regulatory networks in ocular neovascularization

**DOI:** 10.3389/fgene.2022.970224

**Published:** 2022-09-02

**Authors:** Qiang Hu, Xue Zhang, Minghao Sun, Bo jiang, Zhongyu Zhang, Dawei Sun

**Affiliations:** Department of Ophthalmology, The Second Affiliated Hospital of Harbin Medical University, Harbin, China

**Keywords:** ocular neovascular, epigenetic regulation, non-coding RNAs, RNA modification, DNA methylation, histone modifications

## Abstract

Neovascularization is one of the many manifestations of ocular diseases, including corneal injury and vascular diseases of the retina and choroid. Although anti-VEGF drugs have been used to effectively treat neovascularization, long-term use of anti-angiogenic factors can cause a variety of neurological and developmental side effects. As a result, better drugs to treat ocular neovascularization are urgently required. There is mounting evidence that epigenetic regulation is important in ocular neovascularization. DNA methylation and histone modification, non-coding RNA, and mRNA modification are all examples of epigenetic mechanisms. In order to shed new light on epigenetic therapeutics in ocular neovascularization, this review focuses on recent advances in the epigenetic control of ocular neovascularization as well as discusses these new mechanisms.

## 1 Introduction

**FIGURE 1:**
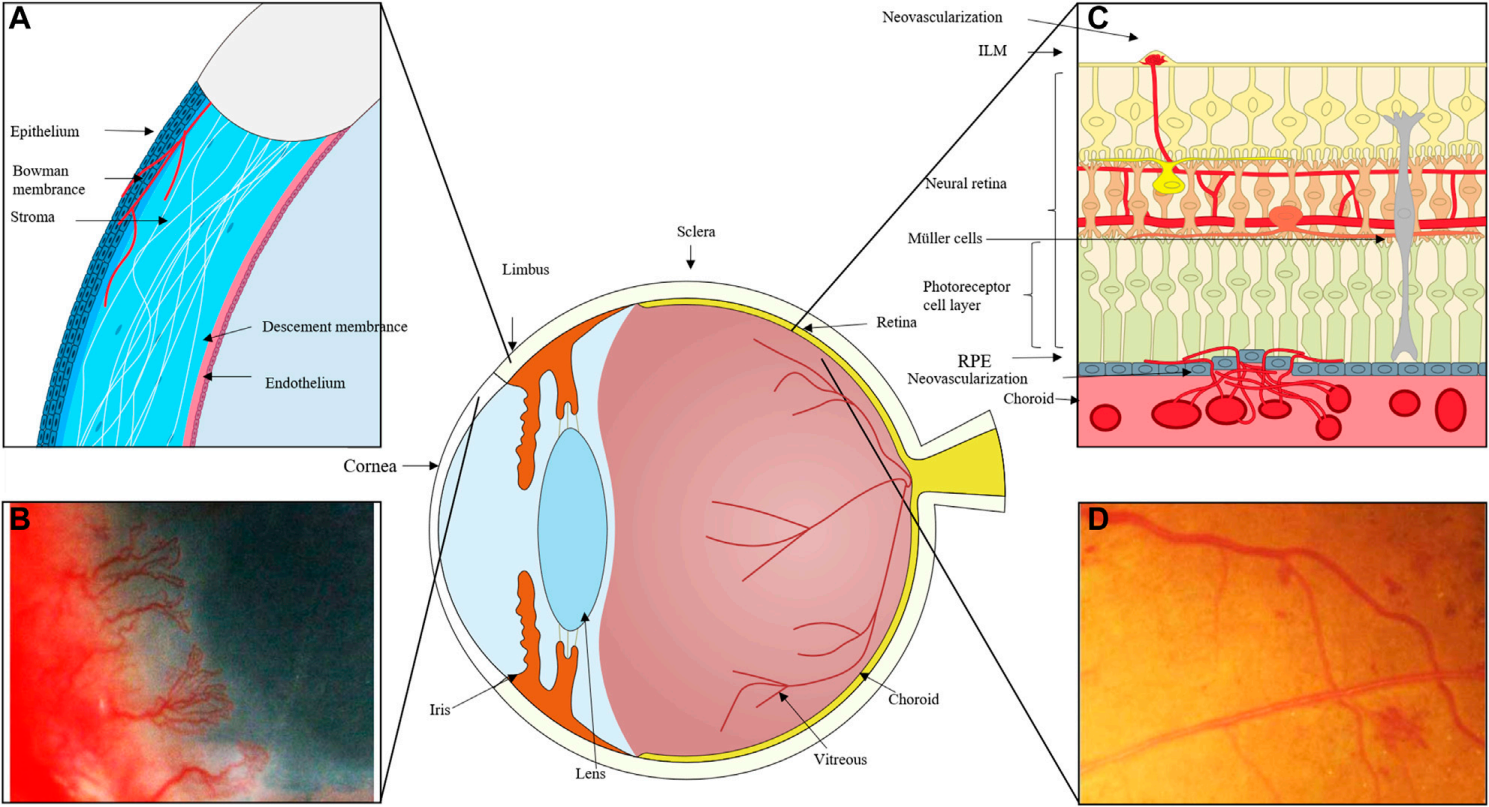
**(A)** The cornea can become damaged by inflammation, trauma, and other factors, and the process of corneal repair is accompanied by the development of neovascularization. New blood vessels emerge from the corneal limbal vessels, and the corneal endothelium proliferates and migrates, growing into the corneal stroma to create corneal neovascularization, which ultimately affects vision. **(B)** Massive corneal revascularisation, affecting the patient’s vision. **(C)** In cases of retinal inflammation, ischemia, and damage to retinal homeostasis, retinal microvascular endothelial cells migrate to the damaged area and, with the help of pro-angiogenic cells like Müller and microglia, secrete pro-angiogenic factors, eventually forming new neovascular cavities. In extreme cases, these neovascular cavities can even break through the inner boundary membrane and grow into the vitreous cavity. When the choroid is exposed to stimulating circumstances like hypoxia and ischemia, choroidal microvessels expand in the direction of these locations, occasionally piercing the RPE layer. **(D)** Fundus photography of neovascularisation in the eye.

Angiogenesis (the formation of new blood vessels) is a physiological and pathological condition required for embryologic development, wound closure, degenerating tissues, and abnormal vascular proliferation. Corneal infection or injury, retinopathy of prematurity (ROP), proliferative diabetic retinopathy (PDR), and age-related macular degeneration (AMD) all-cause corneal neovascularization, retinal neovascularization (RNV), and choroidal neovascularization (CNV), respectively (Figure 1). Vision loss can occur suddenly and irreversibly in many disorders due to ocular neovascularization, or aberrant new blood vessels. Surgery, laser photocoagulation, hormone therapy, and anti-vascular endothelial growth factor (VEGF) medications are currently the main therapeutic options for DR. These therapies, meanwhile, have the potential to be harmful to one’s health or even life and can only temporarily relieve a condition’s symptoms rather than curing it entirely. As a result, it is critical for vision loss control and pharmacological treatment to investigate the molecular mechanisms of neovascularization.

## 2 Angiogenesis and neovascularization

Angiogenesis is one of the side effects of vascular injury. When the vasculature is exposed to negative factors, a variety of cells, including endothelial cells, neo-angiogenic immune cells, endothelial progenitor cells (EPCs), and others, become involved in angiogenesis. The newly developed and unstable vascular types are primarily twofold. On the one hand, endothelial cell proliferation, migration, and capillary tube development can attract circulating endothelial progenitor cells to a damaged region (Potente, Gerhardt, and Carmeliet 2011). Finally, the pericytes cover the endothelial cells, forming a solid vascular system. Similar processes of pathogenic neovascularization exist. Corneal neovascularization primarily arises from the corneal limbal vessels and grows into the layers of the cornea; retinal neovascularization frequently arises from the retinal vessels, breaks through the inner limiting membrane (ILM), and grows into the vitreous; neovascularization of the choroid occurs as a result of the formation of new blood vessels; often, choroidal neovascular membranes (CNVs) form when blood vessels from the choroid penetrate the retinal pigment epithelium and proliferate (Campochiaro, 2000; Nicholas and Mysore 2021). This paper focuses on the impact of dysregulation of epigenetic molecular mechanisms in endothelial cells on neovascularization because it is currently generally accepted that the mechanism of ocular neovascular disease is primarily related to the abnormal biological activity of ocular vascular endothelial cells.

## 3 Epigenetic modifications of ocular neovascularization

An epigenetic modification is a heritable transcriptional change that is not induced by DNA sequence changes (Felsenfeld 2014). Non-coding RNAs like miRNA, LncRNA, CircRNA, and transcription factors have been discovered to have a role in epigenetic regulation of gene transcription and protein modifications in recent years (Tough et al., 2016). Furthermore, as more and more RNA modifications are discovered and recognized, epigenetic regulation is becoming increasingly complex (Frye et al., 2018; Wiener and Schwartz 2021). Epigenetic alterations appear to play a key role in the regulation of ocular neovascularization, according to a growing body of research (Xu M et al., 2018; Lu et al., 2021). DNA methylation, covalent histone modification, noncoding RNA (ncRNA) regulation, and transcription factor regulatory network are the four major epigenetic mechanisms (Tough et al., 2016). DNA methylation (5-methylcytosine, 5mC) is one of the most common types. Histone alterations, such as acetylation and methylation, are important epigenetic factors. Through acetylation, methylation, phosphorylation, and ubiquitylation, the N-terminus of histones is now recognized as a common posttranslational alteration that controls chromatin status and, in turn, leads to aberrant gene regulation (Bates 2020). Studies have found that histone H2AX phosphorylation is essential for retinal neovascularization (Economopoulou et al., 2009). Non-coding RNAs (ncRNAs), including as miRNAs, lncRNAs, and circRNAs, regulate gene expression in a number of ways. A increasing body of evidence suggests that ncRNAs influence gene expression via histone modifications or a combination of histone modification and methylation (Slack and Chinnaiyan 2019).

**FIGURE 2:**
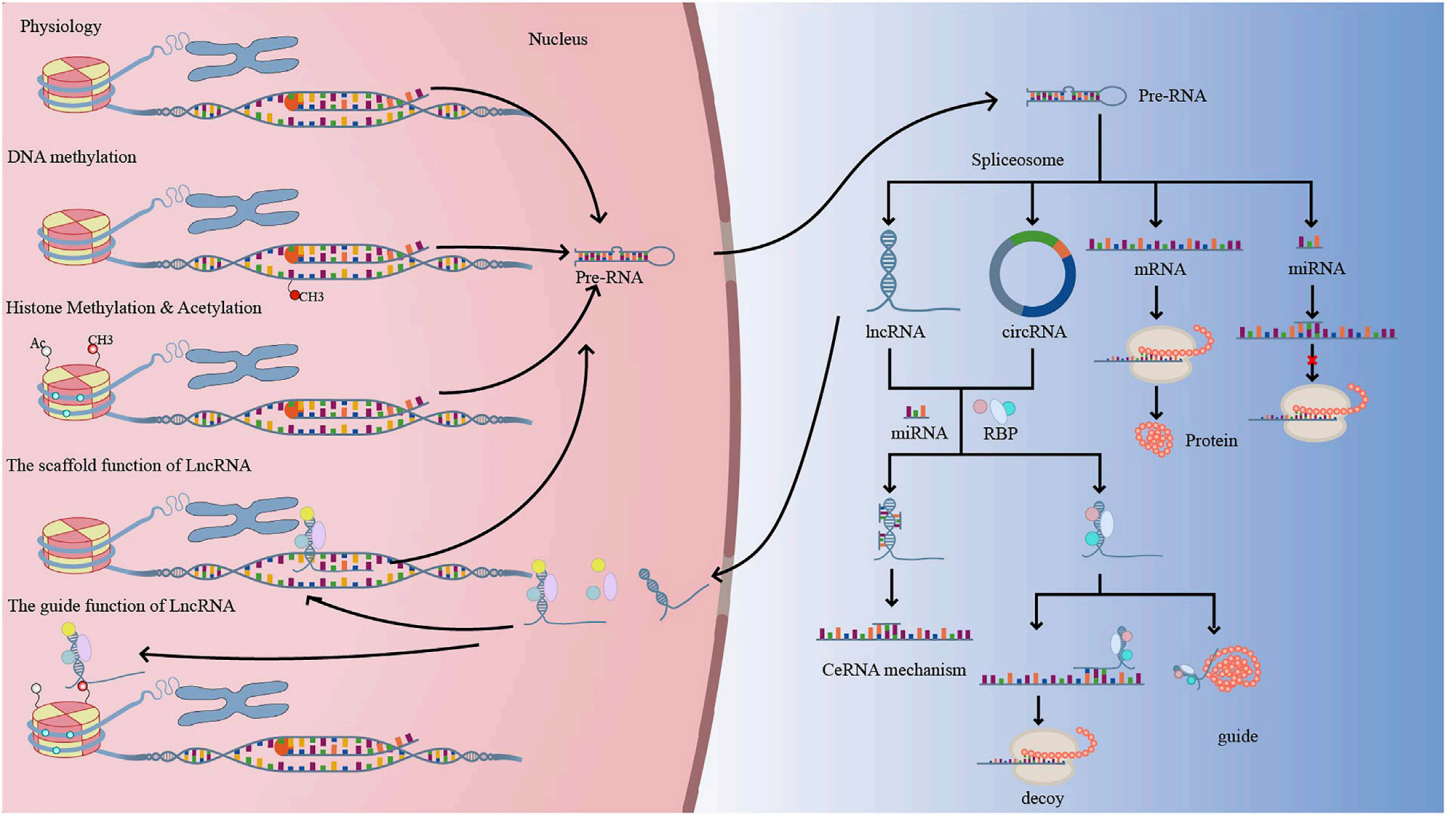
Under physiological conditions, DNA is translated into precursor RNA (pre-RNA), which exits the nucleus and is spliced into protein-coding RNA (mRNA) and non-coding RNA (miRNA, LncRNA, CircRNA, etc.). Normally, when DNA is methylated, pre-RNA transcription decreases, which in turn regulates genes related to neovascularization. LncRNA or CircRNA have similar mechanisms of action, with LncRNA acting as signal, decoy, guide, and scaffold to regulate gene transcription, translation, and post-translational modifications. Methylation and acetylation of histones can alter chromatin accessibility and facilitate transcriptional complexes into promoter regions, regulating gene transcription.

DNA methylation, histone changes, including acetylation and methylation, and ncRNAs have all been linked to the development of neovascularization. The formation of lymphatic and endothelial arteries is connected to DNA methylation, histone 3 lysine 4 methylation (H3K4me), and histone 3 lysine 27 (H3K27me) (Tacconi et al., 2021). Furthermore, neovascularization is aided by the N6-methyladenosine (m6A) modification (Shan et al., 2020). The creation of a complex interaction network between non-coding RNAs and proteins, with a focus on common signaling pathways including VEGF, HIF, Notch, Wnt, and PI3K/AKT. We describe current advances in our understanding of angiogenesis regulatory mechanisms, including as DNA methylation, histone modifications, and the influence of noncoding RNAs on histone or DNA modifications, in this study. The function of epigenetic regulation in the creation of corneal, retinal, and choroidal neovascular membranes could lead to a new therapy option for neovascularization patients (Figure 2).

### 3.1 DNA methylation in neovascularization

The covalent binding of a methyl group to the 5-position of cytosine in DNA happens frequently in sections bigger than 200bp with a high ratio of G + C-rich regions, resulting in the synthesis of 5-methylcytosine (5mC) (Hellebrekers, Griffioen, and van Engeland 2007). DNA methylation, which is performed by DNA methyltransferases (DNMTs), regulates chromatin structure and gene expression by transferring a methyl group from S-adenosylmethionine (SAM) to the fifth carbon of cytosine in CpG patterns (Hellebrekers, Griffioen, and van Engeland 2007; Shafabakhsh et al., 2019). DNMT1, DNMT3a and DNMT3b, and DNMT3L are the four main enzymes that mediate DNA methylation (Shafabakhsh et al., 2019). Distinct methyltransferases serve different purposes. DNMT1 is involved in DNA methylation maintenance, DNMT3A and DNMT3B are required for *de novo* methyltransferases, and DNMT3L interacts with DNMT3A and DNMT3B and dramatically increases their catalytic activity (Yuan et al., 2014). MBD, a DNA methylation binding protein, is principally engaged in DNMT’s execution of DNA methylation functions. MBD1, MBD2, MBD3 and MBD4 are the four methyl-CpG binding domain proteins (Banerjee and Bacanamwo 2010). MBD2, a methyl-CpG-binding protein that regulates gene expression and angiogenesis in vascular endothelium, is one of them. MBD2 regulates angiogenesis in a DNA methylation-dependent mechanism in response to tissue ischemia (Cooper and Keaney 2011). Furthermore, MBD2 can be coupled with a methylation area found in the promoters of endothelial nitric oxide synthase (eNOS) and vascular endothelial growth factor (VEGF) receptor 2 (VEGFR2) in vascular endothelial cells, reducing angiogenesis expression (Rao et al., 2011). Adenosine kinase (ADK) can also influence the methylation state of the VEGFR2 promoter region and promote neovascularization (Xu et al., 2017). In human umbilical artery DNMT3A has been shown to interact with E2F Transcription Factor 1 (E2F1) and control neovascularization-related genes such as the VEGF family, EPhinB2, and HEY2. However, the interaction of DNMT3A with E2F1 that modulates the gene’s transcriptional repression may be independent of the methyltransferase activity (Su et al., 2020).

The alteration of ocular neovascularization was caused by a DNA methylation mechanism (Table1). In an animal model of corneal alkali burn, corneal neovascularization is inhibited by a DNA demethylation alteration involving the PI3K/Akt/mTOR signaling pathways (Li J et al., 2021). The diabetic retinal vasculature is affected by abnormal DNA methylation modifications (Miao et al., 2020).The hypermethylated genes were mostly found in the PI3K/AKT signaling pathway and the extracellular matrix expression protein-related pathway, both of which are beneficial for neovascularization during PDR (Miao et al., 2020). Furthermore, in a hyperglycemic environment, IL-6 can enhance gene expression related with angiogenesis by lowering the protein levels of DNMT1 and DNMT3b (Balakrishnan et al., 2018). On the one hand, hypoxia increased HIF-1 binding site (HBS) methylation, decreased HIF-1 DNA binding, promoted erythropoietin expression, and induced revascularization growth (Wenger et al., 1998); on the other hand, hypoxia can induce HBS demethylation of the VEGF gene promoter, increase HIF-1 on the binding properties of VEGF gene promoters, promote hypoxic induced VEGF gene transactive and VEGF accumulation (Pisani et al., 2018). In neovascular AMD, or early AMD, DNA methylation has shifted (Oliver et al., 2015; Shen et al., 2020). Hypoxia boosted the synthesis of interleukin 17 receptor C (IL-17RC) mRNA in hypoxia-induced retinal pigment epithelium (RPE) cells by boosting the demethylation of the IL-17RC promoter; promoting choroidal neovascularization by a synergistic action of IL-17RC and VEGF (Alivand, Sabouni, and Soheili 2016).

**TABLE 1:** DNA methylation is involved in the regulation of ocular neovascularization.

Types of modification		Functional effects	Effect on ocular neovascularization	References
DNA methylation	Corneal neovascularization	PI3K/Akt/mTOR	Promotion	Miao et al. (2020)
	Retinal neovascularization	HIF-1、eNOS	Promotion	(Wenger et al., 1998; Pisani et al., 2018)
	Retinal neovascularization	IL-6 reduces neovascularization-related gene promoter methylation levels by decreasing DNMT1 and DNMT3B expression	Inhibition	Balakrishnan et al. (2018)
	Choroidal neovascularizati-on	IL-17RC promoter	Promotion	Alivand et al. (2016)

### 3.2 Histone methylation regulates neovascularization

A DNA and protein polymer made primarily of histone proteins, chromatin is the physiological form of the eukaryotic genome. Chromatin remodeling and gene transcription are significantly influenced by histones and their post-translational modifications (Venkatesh and Workman 2015). The accessibility of chromatin may be directly impacted by histone modifications that change the chemistry of the residues that make up their changed forms. By altering the accessibility of RNA polymerase and transcription factors to genes, and notably promoters, chromatin compactness controls transcription at the level of the chromatin (Levy et al., 2017).Histone methyltransferases and demethylases regulate histone methylation. Specific histone tail methylation is frequently linked to transcriptional activation or repression (Choi et al., 2020). Trimethylation or dimethylation of H3K4, H3K36 and H3K79 is generally associated with gene activation, but histone H3 K9 methylation is generally associated with repression of gene expression (Chen et al., 2020). In association with polycomb complex, H3K27me3 acts as a repression histone mark (Agger et al., 2007).Histones can undergo demethylation as well as methylation, for example, H3K27me3 can be removed by the demethylase ubiquitously transcribed tetratricopeptide repeat X (UTX) (Agger et al., 2007). JHDM2A, JHDM1, Lid2, and the lysine demethylase (KDM) family are examples of proteins with the JmjC domain (Tsukada et al., 2006; Yamane et al., 2006; Li et al., 2008; Wang et al., 2017). Furthermore, non-dependent JMIC domain proteins, such as LSD1, can engage in the de-methylating modification of histones (Culhane and Cole 2007). These methyltransferase/demethylated enzymes, as well as other epigenetic genetic regulators, can play a role in neovascularization control (Table 2).

**TABLE 2:** Histone modifications are involved in the regulation of ocular neovascularization.

Types of modification		Functional effects	Effect on ocular neovascularization	References
Histone Methylation	Retinal neovascularization	HES-1	Promotion	Choi et al. (2020)
		Histone methylation modifications regulate neovascularization by acting on HIF-1α, VEGF, Jagged1 and other signaling pathways		
	Corneal neovascularization	Regulation of FoxO3a and P13K/AKT signal axis	Promotion	Wan et al. (2020)
Histone acetylation	Retinal neovascularization	Acts on NF-κB or VEGF to regulate neovascularization		(Aurora et al., 2010; Fu et al., 2014; Yuan et al., 2014)
		Increased levels of histone acetylation within PRAGM promote angiogenesis.		Liu X et al. (2020)
	Corneal neovascularization	Promotes the expression of genes related to neovascularization, such as VEGF.	Promotion	(Zhou et al., 2014b; Zhou Q et al., 2014)
	Choroidal neovascularization	Promotes neovascularization by regulating the levels of histone H3, H4	Promotion	Dahbash et al. (2019)

A number of histone changes are involved in the process of neovascularization. SETD8 is an H4K20me mono-methyltransferase. SETD8 was shown to have a role in angiogenesis in human umbilical vein endothelial cells (HUVEC) in part through influencing HES-1; in the meantime, SETD8 inhibitors suppressed abnormal neovascularization in the mouse oxygen induced retinopathy (OIR) model (Choi et al., 2020). Enhancer of zeste homolog 2 (EZH2), a major catalytic component of polycomb repressive complex 2 (PRC2), regulates the process of neovascularization by catalyzing the trimethylation of histone H3K27 (Ohtani et al., 2011). The expression of ROS-dependent activation of the Forkhead-box protein O3a (FoxO3a) pathway and P13K/AKT signaling is reduced when EZH2 is inhibited, lowering corneal neovascularization (Wan et al., 2020). PHD finger protein 8 (PHF8), a histone demethylase, may regulate HIF1 and H3K4me3 signaling to govern neovascularization. PHF8 upregulates HIF-1 and activates histone demethylase KDM3A in anoxic settings, which maintains H3K4me3 levels and upregulates HIF1 downstream target genes (Maina et al., 2017). VEGF stimulates angiogenesis by increasing the expression of EZH2 in endothelial cells (Lu et al., 2010). Furthermore, VEGF inhibited the production of EZH2 by downregulating miR-101 expression and therefore promoting angiogenesis (Smits et al., 2011). JARID1B occupies and decreases histone 3 lysine 4 methylation levels on the HOXA5 promoter, then inhibits HOXA5 production to retain angiogenic capacity (Fork et al., 2015). MLL is a histone H3 lysine 4 (H3K4) methyl transferase (HMT) that may methylate H3K4 at HOX9 promoters, increasing neovascularization via influencing endothelial cells sprout and migration (Diehl et al., 2007). Furthermore, LSD1-mediated demethylation of HIF-1a under hypoxic circumstances leads to HIF-1a stability, which promotes the establishment of neovascularization (Kim et al., 2016). In DR, LSD1 has a role in the neovascularization of endothelial cells, which is triggered by high blood glucose levels (Zhao et al., 2020). Increased expression of Jagged1 by histone lysine-specific demethylase 2A (KDM2A) might enhance neovascularization (Chen et al., 2016). JHDM1D (also known as KDM7A) is a histone demethylation enzyme that may change histone methylation levels and induce neovascularization by controlling the expression of VEGF-A (Osawa et al., 2011).

### 3.3 Histone acetylation in neovascularization

Histone acetyltransferase (HAT) and histone deacetylase (HDAC) regulate histone acetylation in a dynamic manner (Aikawa et al., 2006; Park and Kim 2020). Histone acetylation happens mostly on its histone tail, which can activate or repress gene transcription. When histone lysine is acetylated, the nucleosome’s histone structure changes from compact to loose, which increases RNA polymerase accessibility and hence increases gene expression (Park and Kim 2020). Histone deacetylation causes transcriptional repression by decreasing the DNA-histone link (Buysschaert et al., 2008). Histone acetylation is mostly related with the GNAT, MYST, and p300/CBP families (Xia et al., 2020). Histone deacetylation is primarily mediated by two protein families: the histone deacetylase family (HDAC1-11) and the Sirtuin protein family (SIRT1-7) (Portela and Esteller 2010; Park and Kim 2020). Dynamic modifications of histone acetylation have been shown to play an important role in neovascularization, with HDAC6, HDAC7, and HDAC9 being positive regulators of angiogenesis and enhancing endothelial cell angiogenesis, whereas HDAC5 inhibits endothelial cell sprouting by suppressing the expression of angiogenic guidance factors (Kaluza et al., 2013).

Histone acetyltransferase P300 dramatically increases MRTF-A transcriptional activity on VE-cadherin genes by increasing acetylation of H3K9, H3K14, and H4, hence promoting vascular development and angiogenesis (Shu et al., 2015). This is similar to previous studies in that neither SRF nor MRTF-A have intrinsic histone acetylase activity, but they can promote chromatin remodeling directly or indirectly through ribosomal reorganization; histone recruitment to p300/CBP can be promoted during chromatin remodeling, increasing the expression of angiogenesis-related genes (Hanna et al., 2009).The VEGF promoter region is efficiently upregulated by H3K9 acetylation, triggering VEGF-induced angiogenesis (Fu et al., 2014). Under high glucose conditions, histone acetyltransferases CBP/p300 and p/CAF acetylate histone H3 (K9, K14) and H4 (K8, K8, K12) and increase NF-κB expression, whereas NF-κB and HAT recruitment synergistically regulate chromatin remodeling and promote the expression of inflammation and angiogenesis-related genes (Miao et al., 2004; Aurora et al., 2010). Furthermore, in early DR, a high-glucose environment decreases the amount of Sirt6 in Müller cells while increasing acetylation of H3K56, resulting in reduced levels of neuroprotective factor (BDNF) protein and greater levels of VEGF protein, encouraging early neurodegenerative lesions and vascular dysfunction (Zorrilla-Zubilete et al., 2018). It was recently discovered that high levels of glycolysis in macrophages/microglia produce a lot of acetyl coenzyme a, which leads to histone acetylation and pathological retinal angiogenesis-associated glycolytic macrophages/microglia (PRAGM)-associated gene induction, which reprograms macrophages/microglia to an angiogenic phenotype and promotes retinal pathological angiogenesis (Liu C et al., 2020). HDAC controls the expression of HoxA9, which affects the expression of endothelial characteristic genes including endothelial nitric oxide synthase, VEGFR2 and VE-cadherin, promoting the differentiation of adult histocytes to endothelial cells and engaging in neovascularization. HoxA9 expression is decreased when HDAC inhibitors are employed, which decreases neovascularization (Kaluza et al., 2013).HDAC enhances the production of pro-angiogenic genes VEGF, β-FGF, and TGFβ1 in a corneal alkali burn model, promoting angiogenesis, whereas inhibitors limit neovascularization (Zhou et al., 2014a; Zhou et al., 2014b). The HDAC inhibitor AN7 inhibits CNV progression by antagonizing the deacetylation activity of class I and II HDAC, resulting in hyperacetylation of histones H3 and H4, as well as downregulating the production of pro-angiogenic factors VEGF and fibroblast growth factor (FGF-2) (Dahbash et al., 2019). These findings imply that histone acetylation regulation is involved in angiogenesis and that histone deacetylase inhibitors are employed to treat ocular neovascularization, although the molecular mechanisms of ocular neovascularization remain unknown.

### 3.4 Epigenetic modifications of non-coding RNAs and ocular neovascularization

These results in light of earlier research on the diversity of non-coding RNAs led to the classification of ncRNAs into two main groups: structural non-coding RNAs and regulatory non-coding RNAs.The rRNA and tRNA subgroups of structural non-coding RNAs.Regulatory non-coding RNAs are further divided into three classes, small, medium and long non-coding RNAs (Nagano and Fraser 2011). Small ncRNAs include miRNAs, tsRNAs, and piRNAs, but big ncRNAs are mostly subclasses like LncRNAs, CircRNAs, and pseudogenes. Furthermore, many more are categorized as medium non-coding RNA with a size between 50–200 nucleotides, including snoRNA, prompts, tiRNA, snRNA, and many more. (Dahariya et al., 2019; Slack and Chinnaiyan 2019). This research focuses on the interactions between these three RNAs and epigenetics as miRNA, LncRNA, and CircRNA are more extensively explored in ocular neovascularization.

#### 3.4.1 Epigenetic modification of MiRNA and ocular neovascularization

MicroRNAs are a kind of small ncRNA with a length of around 22 nucleotides that are mostly generated through RNA pol II transcription (Slack and Chinnaiyan 2019; Liu Z et al., 2020). MiRNA regulates the expression of genes involved in angiogenesis by binding to the 3′UTR region in mRNA through its own 5′ end (Liu X et al., 2020). The emphasis of this paper is on the epigenetic control of miRNAs that are related with ocular neovascularization (Table 3).

**TABLE 3:** Potential epigenetic regulatory networks involving ocular neovascular miRNAs.

miRNAs	Types of modification	Functional effects	Effect on ocular neovascularization	References
miR-126	DNA Methylation	DMNT1 can bind miR-126 promoter to regulate miR-126 expression	Overexpression of miR-126 inhibits ocular neovascularization	Xue et al. (2020)
	Histone methylation	The histone methylation enzyme MMSET induces altered levels of histone methylation in the promoter region of miR-126 and suppresses the expression of miR-126.		Min et al. (2013)
MiR-150	DNA Methylation	miR-150-5p promoter methylation and reduced miR-150-5p expression9		Hu et al. (2019)
	Histone methylation	Histone methylation transferase, which blocks the processing of miR-150 precursors into mature miRNAs via the MYC/LIN28 functional axis, reduces miR-150 expression.		Jiang et al. (2012)
	Histone Acetylation	The miR-150 promoter can be silenced by SIRT1, leading to abnormal levels of p53 acetylation and activation of the AMPK signaling pathway.		(Dong et al., 2021)
miR-29	DNA Methylation	miR-29 promoter methylation is negatively correlated with its expression, suggesting that DNA methylation can inhibit miR-29 expression.	Inhibits ocular neovascularization	Wang et al. (2020)
	Histone methylation	miR-29 regulates histone or DNA methylation levels and promotes chromosome repair.		(Heid et al. 2017; Lyu et al. 2018)
	Histone Acetylation	Deacetylation of histones in the miR-29 promoter region leads to upregulation of miR-29		Amodio et al. (2016)
miR-200	DNA Methylation	DNA methylation-related enzymes aberrantly methylate the miR-200 promoter and inhibit MiR-200b expression	Overexpression of miR-200 inhibits ocular neovascularization	(Ning et al. 2015; Singh et al. 2017)
	Histone methylation	PKC or EZH2-mediated histone methylation suppresses MiR-200 expression		(Ning et al., 2015; Ruiz et al. 2015)
MiR-21	DNA Methylation	Abnormal methylation of promoter DNA due to different stimuli leads to abnormal MiR-21 expression and promotes abnormal endothelial cell function	Elevation of miR-21 promotes angiogenesis	(Lu et al., 2020; Penaloza et al. 2020)
	Histone Acetylation	AKT2 mediates transcriptional regulators such as CBP/p300 to induce altered levels of histone acetylation and regulate miR-21 expression.		Polytarchou et al. (2011)
miR-24	Histone methylation	Demethylation of H3K27 leads to downregulation of miR-24	miR-24 inhibits ocular neovascularization	Ni et al. (2019)
MiR-34a	Histone Acetylation	Regulation of histone acetylation levels through modulation of Sirt1	MiR-34a inhibits ocular neovascularization	Arunachalam et al. (2016)

##### 3.4.1.1 MiR-126

MiR-126 governs the formation of different retinal vascular layers as well as ocular neovascularization (Zhao et al., 2015; Martinez and Peplow 2021). MiR-126-3p promotes immature capillary development by decreasing Sprouty-related protein1 (SPRED1) and phosphoinositol-3 kinase regulatory subunit 2 (PLK2) expression, boosting ERK phosphorylation, and modulating the mobility of endothelial cell (EC)-based membrane-like complex structures and stabilizing networks (Pitzler et al., 2016). Furthermore, MiR-126 promotes the differentiation of pluripotent stem cells into retinal ganglion stem cells and the release of VEGF, which in turn regulates retinal neovascularization (Ye et al., 2018). MiR-126 epigenetic regulation is also implicated in the process of neovascularization. The miR-126 promoter’s methylation is important in the control of neovascularization (Zhang et al., 2013; Xue et al., 2020). High hyperglycemia triggers human umbilical vein endothelial cell (HUVEC) endogenous H2S-DMNT1-miR-126 regulatory axis, causing endothelial cell migratory abnormalities (Xue et al., 2020). Histone methyltransferase (MMSET) can construct chromatin modification complexes by binding to the miR-126 promoter, KAP1 corepressor, and histone deacetylase, resulting in increased H3K9 trimethylation and reduced H3 acetylation and reducing miR-126 production (Min et al., 2013). According to the findings, epigenetic regulation involving miR-126 has the potential to be a molecular target for the therapy of ocular neovascularization in the future.

##### 3.4.1.2 MiR-150

MiR-150 has been established in studies to perform a significant regulatory function in pathological neovascularization of the retina and choroid (Shen et al., 2008; Liu et al., 2016; Chen et al., 2019). MiR-150 targets stearoyl coenzyme A desaturase-2 in human peripheral blood mononuclear cells to coordinate macrophage-mediated inflammation and pathological angiogenesis in AMD in a VEGF-independent way (Lin et al., 2018). MiR-150 suppresses endothelial cell proliferation, migration, and tube formation while also specifically reducing the expression of different angiogenic regulators such as CXCR4, DLL4, and FZD4 in endothelial cells, hence limiting pathogenic ocular neovascularization (Liu et al., 2015). Mixed lineage leukemia gene (MLL) histone methylation transferase, which negatively controls miR-150 by preventing miR-150 precursors from being processed into mature miRNAs via the MYC/LIN28 functional axis (Jiang et al., 2012). Furthermore, it was discovered that the DNMT1 enzyme methylates CpG islands in the miR-150-5p gene promoter (Hu et al., 2019). These findings imply that miR-150 epigenetic control may play a role in the process of ocular neovascularization.

##### 3.4.1.3 MiR-29

MIR-29b-3p has been demonstrated to adversely influence the production of VEGF A and platelet-derived growth factor B (PDGF B), as well as the proliferation and angiogenesis of retinal microvascular endothelial cells (Tang et al., 2020). Furthermore, in laser-induced choroidal neovascularization, activation of NF-κB suppressed MiR-29 expression and increased choroidal neovascularization development (Cai et al., 2014). In human cell lines, miR-29 regulates the gene expression of DNMT1, DNMT3a, Dnmt3b, SIRT1, SIRT3, and ROS, as well as overall DNA methylation and protein acetylation levels (Morita et al., 2013; Sonkar, Kay, and Choudhury 2019). MiR-29 reduce the abundance of H4K20me3 and the degree of DNA methylation, decreasing DNA damage repair and the expression of genomic signatures (Heid et al., 2017; Lyu et al., 2018). Furthermore, DNA methylation and histone changes can regulate miR-29 family control and hence neovascularization. HDAC4 silencing causes hyperacetylation of the miR-29b promoter, resulting in increased miR-29b expression (Amodio et al., 2016). These findings imply that molecular pathways involved in epigenetic interactions might cause abnormal miR-29 dysregulation and mediate angiogenesis.

##### 3.4.1.4 MiR-200

MiR-200b/c protects human retinal microvascular endothelial cell (hRMECs) from high-glucose-induced dysfunction by decreasing vasohibin-2 (VASH2) and decreases endothelial cell migration and proliferation (Ding et al., 2017). Furthermore, hypoxia decreases miR-200b expression and increases Ets-1 expression in endothelial cells, promoting angiogenesis (Chan et al., 2011). The transcriptional regulation of microRNA-200 family members is regulated by DNMT1/DNMT-3A and histone methyltransferase (EZH2) (Ning et al., 2015). High glucose levels reduced the expression of the endothelial cell DNA methylation transferases DNMT1 and DNMT3A, resulting in miR-200b promoter hypermethylation and regulating neovascularization (Singh et al., 2017). Furthermore, high glucose stimulation inhibits miR-200b production by activating PRC2-mediated histone methylation, which lowers VEGF expression and increases retinal inflammation and neovascularization (Ruiz, Feng, and Chakrabarti 2015). MiR-200b is also a part of a complex regulatory network that includes LncRNAs and epigenetically linked proteins. Yes-associated protein (YAP1), which regulates the MALAT1/miR-200b-3p/VEGFA axis, may contribute to retinal neovascularization in DR lesions (Han et al., 2020).

##### 3.4.1.5 MiR-21

MiR-21 is a critical regulator of ocular neovascularization (Guduric-Fuchs et al., 2012; Merrigan and Kennedy 2017; Vivanco-Rojas et al., 2020). By modulating the expression of its target protein Maspin, miR-21-5p affects the PI3K/AKT and ERK signaling pathways, and hence diabetic retinal neovascularization (Qiu et al., 2018). MiR-21 regulates and targets PPARa, promoting OIR retinal neovascularization and inflammatory responses (Chen et al., 2017). Chronic hypoxia and high glucose levels can cause abnormal methylation of the miR-21-5p promoter, which in turn controls eNOS production and causes endothelial dysfunction (Penaloza, Soto-Carrasco, and Krause 2020). Under hypoxic circumstances, intracellular Akt2 can control miR-21 production and hence neovascularization via NF-κB, CREB, and CBP/p300 binding to the miR-21 promoter and regional acetylation of histone H3K9 (Polytarchou et al., 2011).

##### 3.4.1.6 Others

In mice, miR-24 suppresses laser-induced choroidal neovascularization (Zhou et al., 2014a). The study discovered that miR-24 is engaged in epigenetic control; UTX on chromosome X dramatically decreases methylation levels in the miR-24 promoter via histone H3K27 demethylase (KDM6) (Ni et al., 2019). However, it has to be shown if this epigenetic control is present in ocular neovascularization. MiR-34a also influences retinal angiogenesis via the Notch1/VEGF signaling pathway (Shi S et al., 2019). Furthermore, via modulating Sirt1 in hyperglycemia-stimulated microvascular endothelium, miR-34a might be implicated in neovascularization in diabetic vasculopathy (Arunachalam et al., 2016). These miRNAs play important physiological roles in neovascular regulation, and their involvement in the formation of complex networks of epigenetic regulation is becoming more apparent; understanding the mechanisms of formation of these abnormal miRNAs is critical for preventing and controlling neovascularization.

#### 3.4.2 Epigenetic modifications of LncRNA and ocular neovascularization

LncRNA has a wide range of biological roles, including regulating pathological neovascularization of the cornea, retina, and choroidal disorders (Huang et al., 2015; Zhang C et al., 2020). Depending on their subcellular location, lncRNAs have diverse regulation mechanisms, according to studies. By binding to RNA-binding proteins, LncRNAs in the nucleus impact histone modifications, DNA methylation status, and maturation of certain precursor mRNAs, which ultimately govern gene transcription and protein production by regulatory transcription or post-transcriptional regulation (Xu Z et al., 2018). LncRNAs in the cytoplasm frequently pass through sponge target miRNAs and control intracellular signaling via competing endogenous RNAs (CeRNA) regulation mechanisms (Xu M et al., 2018; Ghafouri-Fard et al., 2020). The control of neovascularization relies heavily on these regulatory systems. The epigenetic regulatory mechanisms implicated in LncRNAs related with ocular neovascularization in recent years are the emphasis of the following paragraphs (Table 4).

**TABLE 4:** Potential epigenetic regulatory networks involving ocular neovascular LncRNAs.

LncRNAs	Types of modification	Functional effects	Effect on ocular neovascularization	References
NEAT1	DNA methylation	DNMT3A decreases NEAT1 promoter methylation levels and promotes NEAT1 expression	Overexpression of NEAT1 promotes ocular neovascularization	Duan et al. (2020)
	Histone methylation	NEAT1 binds to EZH2 and mediates H3K27 trimethylation to activate the Wnt/β-catenin signaling axis		(Chen et al., 2018)
	Histone acetylation	NEAT1 promotes Sirt1 expression by inhibiting miRNA physiological effects through binding to miRNAs		Zhou Z et al. (2019)
H19	Histone methylation	H19 can bind to EZH2 to regulate histone methylation levels and promote angiogenesis.	Promotes ocular neovascularization by regulating the function of macrophages or endothelial cells	(Guo et al., 2018; Yuan et al., 2019)
	DNA methylation	H19 can regulate the promoter methylation level of angiogenesis-related genes directly by binding to EZH2, and also by binding to DNA methylation-regulated related proteins to promote angiogenesis.		(Thomas et al., 2019; Klein et al., 2016)
HOTAIR	Histone methylation	HOTAIR regulates the expression of angiogenesis-related genes and activation of pathways by interacting with histone methylation-regulated related proteins.	Promotes neovascularization	(Biswas et al., 2021)
MALAT1	DNA methylation	MALAT1 is regulated by DNA methylation enzymes and regulates endothelial cell dysfunction and neovascularization	Overexpression of MALAT1 promotes neovascularization	Biswas S et al. (2018)
	Histone methylation	MALAT1 can interact with EZH2 to regulate ocular neovascularization and can also be regulated by histone methylation enzymes that mediate MALAT1 expression.		(Wu et al., 2019; Tee et al., 2014)
MEG3	DNA methylation	The MEG3 promoter undergoes DNA methylation and represses the expression of MEG3.	Overexpression of MEG3 inhibits neovascularization.	He et al. (2021)
	Histone methylation	MEG3 interacts with EZH2 to reduce the recruitment of histone methylation-related enzymes at H3K27 modification sites and inhibit the methylation levels of histones.		Zhou R et al. (2020)
	Histone acetylation	MEG3 regulates endothelial cell dysfunction and angiogenesis in a high-glucose environment by regulating SIRT1 expression.		(Tong et al., 2019)
SNHG1	Histone methylation	SNHG1 can interact with PKC2 to regulate histone methylation levels.	SNHG1 promotes neovascularization	Xu M et al. (2018)
SNHG7	Histone methylation	SNHG7 interacts with EZH2, decreases H3K27 methylation level in DKK1 region and activates Wnt/β-catenin signaling pathway	SNHG7 inhibits neovascularization	Chi et al. (2020)
	Histone acetylation	SNHG7 suppresses miR-543 and promotes Sirt1 expression.		Ke et al. (2019)
Fendrr	Histone methylation	Fendrr increases gene transcription by regulating histone methylation levels.	Increased expression of Fendrr promotes neovascularization.	He et al. (2018)
GATA6-AS	Histone methylation	GATA6-AS promotes angiogenesis in endothelial cells by regulating histone methylation levels through interaction with LOXL2.	Elevation of GATA6-AS promotes neovascularization.	Neumann et al. (2018)

##### 3.4.2.1 NEAT1

In corneal, retinal, and choroidal neovascularization, long noncoding RNA nuclear enriched abundant transcript 1 (NEAT1) has been widely researched. By sponging with miR-377 and boosting the production of VEGFA, SIRT1, and BCL-XL, NEAT1 controls neovascularization (Zhou Y et al., 2019).Furthermore, miR-194-5p has been demonstrated to increase cell invasion and migration by inhibiting methylation of the NEAT1 promoter region by targeting the 3′ UTR of DNMT3A (Duan et al., 2020).

##### 3.4.2.2 H19

In vascularized corneas, H19 is abundantly expressed. By targeting vascular endothelial growth factor-A, lncRNA H19 can increase ocular neovascularization (Sun et al., 2019). In addition, LncRNA H19 may have a role in angiogenesis epigenetic regulation. H19 can bind to EZH2, a histone methyltransferase. H19 improves EZH2’s ability to recruit methyl groups to the promoter region of the angiogenesis inhibitor gene vasopressor-1 (VASH1), inhibiting VASH1 expression and promoting angiogenesis (Yuan et al., 2019); H19 also promotes HIF-1 histone H3K4me3 methylation and increases HIF-1 expression by recruiting EZH2, speeding up fibroblast activation and angiogenesis regulation (Guo et al., 2018). H19 regulates endothelial cell dysfunction by raising histone acetylation upstream of miR-200 and controlling miR-200 production; it also regulates retinal neovascularization by acting on miR-200 in a non-dependent manner (Thomas et al., 2019).

##### 3.4.2.3 HOTAIR

By influencing epigenetic processes such as histone methylation, acetylation, DNA methylation, and transcription factors, HOX transcript antisense intergenic RNA (HOTAIR) facilitates diabetic retinopathy development (Biswas et al., 2021). HOTAIR controls the status of chromatin and gene transcription by functioning as a scaffold for directed transcription and controlling histone methylation changes, among other things (Tsai et al., 2010). HOTAIR inhibits VE-calmodulin transcription while enhancing VEGFA transcription by binding to histone lysine specific demethylase 1 (LSD1) (Zhao et al., 2020). Furthermore, HOTAIR promotes angiogenesis by activating VEGFA transcription directly and upregulating VEGFA and Ang2 expression through glucose regulated protein 78 (Fu et al., 2016). Histone alterations also have a role in the regulation of lncRNA HOTAIR. Under hypoxic circumstances, the HOTAIR promoter is regulated by histone methyltransferase MLL1, histone acetylase p300, and HIF-1α, which are all abundant at the HOTAIR promoter (Bhan et al., 2017). Furthermore, JMJD6, a protein that has both lysyl hydroxylase and arginine demethylase capabilities, binds to the HOTAIR promoter region and stimulates HOTAIR expression through its lysyl hydroxylase/demethylase activity (Biswas A et al., 2018). According to these findings, HOTAIR may be involved in the genetic regulation of related gene expression in neovascularization.

##### 3.4.2.4 MALAT1

Metastasis associated lung adenocarcinoma transcript 1 (MALAT1) is one of the most significant epigenetic regulators in diabetic retinopathy, and it regulates endothelial cell function and angiogenesis (Biswas S et al., 2018). MALAT1 promoter CpG island methylation is affected by high hyperglycemia, which affects endothelial cell function (Biswas A et al., 2018). MALAT1 regulates HIF-1 expression and interacts with nuclear transcription factors to induce neovascularization (Jayasuriya et al., 2020). MALAT1 is abundantly expressed in OIR mice’s retinas, and it promotes RNV development primarily via regulating the CCN1-Akt-VEGFA pathway (Wang et al., 2020). MALAT1 increases angiogenesis in ischemia-reperfusion damage via modulating the 15-lipoxygenase 1/STAT3 signaling pathway (Wang C et al., 2019). MALAT1 blocks the Wnt signaling pathway by suppressing CTNNB1 transcription and expression, which attracts methyltransferases to boost CTNNB1 promoter methylation, primarily in the CTNNB1 promoter region (Li et al., 2019). Histone modifications also have a role in MALAT1 expression. By binding to the promoter of the MALAT1 gene, resulting in the demethylation of histone H3K9 in the promoter region of the MALAT1 gene, the histone demethylase JMJD1A can boost MALAT1 expression, which stimulates cell proliferation and migration (Tee et al., 2014). In addition, JMJD2C protein entering the nucleus improved the -linked protein signaling pathway in colorectal cancer cells by lowering H3K9me3 and H3K36me3 histone methylation levels in the MALAT1 promoter region and upregulating MALAT1 expression (Wu et al., 2019). YAP1 may enhance neovascularization in diabetic retinopathy via the MALAT 1/MiR-200b-3p/VEGFA regulation axis in high-glucose-induced endothelial cells and high-fat diet-induced diabetic mice retina (Han et al., 2020). SIRT6 binds to the MALA T1 promoter region and reduces MALAT1 expression, allowing neovascularization to occur (Qin et al., 2019). However, the researchers did not investigate the impact of SIRT6’s deacetylase activity on MALAT1.

##### 3.4.2.5 MEG3

There are several LncRNAs that inhibit neovascularization, in addition to the potential that the aforementioned LncRNAs promote neovascularization. Maternally expressed gene 3 (MEG3) reduces angiogenesis *in vivo* and *in vitro* by negatively influencing the notch system; lncRNA-Meg3 knockout mice had increased vascular endothelial growth factor pathway gene expression and microvessel density (Kumar and Goyal, 2017; Liu et al., 2017). Furthermore, it has been discovered that DNMT1 increases MEG3 promoter methylation by recruiting methyltransferases to inhibit MEG3 expression (He Y 2021 Mar 1). MEG3 inhibits histone methylation by interacting with EZH2 and lowering EZH2 and H3K27me3 recruitment to EN2 (Zhou R et al., 2020). TTR interacts with Poly(A)-binding protein cytoplasm 1 (PABPC1), which suppresses hREC proliferation and angiogenesis, to enhance lncRNA-MEG3 expression (Fan et al., 2019). These research discovered that MEG3-targeting epigenetic regulatory systems might be one of the treatment options for ocular neovascularization.

##### 3.4.2.6 SNHG1 and SNHG7

In the DR, the lncRNA small nucleolar RNA host gene 1 (SNHG1) is abnormally expressed (Yang et al., 2021). SNHG1 enhances vascular endothelial cell proliferation and angiogenesis in TNF-stimulated HUVEC (Zhang et al., 2020a).SNHG1-mediated histone changes are also implicated in neovascularization. SNHG1 interacts directly with the polycomb repressor complex 2 (PRC2), which regulates cell growth and differentiation by regulating histone methylation of the Kruppel-like factor 2 (KLF2) promoter and cell cycle protein-dependent kinase inhibitor 2B (CDKN2B) in the nucleus (Xu Z et al., 2018; Li, Guo, and Wu 2020). By decreasing miR-543 and increasing Sirt1 expression, SNHG7 prevents high glucose-induced angiogenesis in human retinal endothelial cells (Ke et al., 2019). SNHG7 stimulates the Wnt/-catenin signaling pathway by inhibiting DKK1 production, which primarily controls H3K27 methylation levels by creating a complex with EZH2 in the DKK1 promoter region (Chi et al., 2020). These findings show that epigenetic control of SNHG7 or SNHG1 may play a role in neovascularization.

##### 3.4.2.7 Others

Fendrr, a long noncoding RNA, enhances high glucose-induced proliferation and angiogenesis in human retinal endothelial cells, perhaps through regulating VEGF (Shi Y et al., 2019). Meanwhile, through the miRNA-214-3p/TET2 regulatory axis, LncRNA Fendrr may be involved in the modification of RASSF1 promoter methylation; however, TET2 is an ancient DNA demethylase, suggesting that LncRNA Fendrr indirectly mediates the involvement of DNA demethylases in neovascularization via the CeRNA mechanism (He Z 2018 Oct 15). Through changes in histone methylation, the lncRNA GATA6-AS interacts with the epigenetic regulator LOXL2 to modulate endothelial gene expression for angiogenesis (Neumann et al., 2018). These findings show that, whereas LncRNA-mediated DNA methylation, histone changes, and other modifications in ocular neovascularization have yet to be extensively reported, their precise mechanisms of action may be revealed in the near future.

### 3.5 CircRNA and ocular neovascularization

Circular RNA (circRNA) has been shown to be differentially expressed in corneal, retinal, and choroidal neovascularization, suggesting that it may regulate neovascularization by regulating intracellular signaling molecules such as extracellular matrix production, MAPK cascade signaling, and renin-angiotensin system pathways (Cao et al., 2019; Zhou Z et al., 2019; Zhang et al., 2020b; Liu C et al., 2020). CircRNA, like LncRNA, promotes angiogenesis mostly through the mechanism of sponge miRNA and regulates miRNA target genes. CircFndc3bk in endothelial-derived exosomes may play a crucial role in PDR neovascularization (Li X et al., 2021). CZBTB44 stimulates choroidal angiogenesis by functioning as a miR-578 sponge, decreasing miR-578 activity and increasing production of vascular endothelial growth factor A (VEGFA) and vascular cell adhesion molecule-1 (VCAM1) (Zhou Y et al., 2020). CIRCCRNA-0006896 promotes HUVEC migration and proliferation via the regulatory axis of microRNA-1264/DNMT1 (Wen et al., 2021). More regulatory mechanisms influencing epigenetic inheritance will be revealed in the future as CircRNAs are extensively investigated in ocular diseases.

### 3.6 M6A modifications are involved in ocular neovascularization

A rising number of studies have shown that RNA modifications, such as N6-methyladenosine (m6A) and 5-methylcytidine (m5C), play a significant role in epigenetic regulation illnesses, resulting in full base isomerization and aberrant alterations in RNA activity (Wiener and Schwartz 2021). M6A methylation changes are mediated by writer, eraser, and reader proteins, and these RNA modifying proteins (RMP) play critical roles in RNA stability and translation activity (Frye et al., 2018). Currently, m6A alterations are particularly prevalent in ocular neovascularization. The M6A writing protein METTL3 has been demonstrated to have a pro-angiogenic function in retinal and corneal neovascularization, with the major mechanism being to increase mRNA translation of LRP6 and DVL1 in a YTHDF1-dependent way (Yao et al., 2020). METTL3 knockdown hindered cell proliferation, migration, and capillary formation in HUVEC, with the major mechanism depending on its m6A writing enzyme activity to raise PHLPP2 levels and decrease mTOR phosphorylation, resulting in vascular defects and reduced AKT activation (Parial et al., 2021). Furthermore, FTO, a demethylase involved in m6A modification, has increased expression in sick corneal endothelial cells; increased FTO promotes low m6A methylation levels of the pro-angiogenic gene FAK, resulting in improved FAK stability and neovascularization (Shan et al., 2020). KAT1 can rely on histone acetylase activity to trigger YTHDF2 transcription, which can reduce ITGB1 mRNA stability via M6A modification and limit microvessel density and angiogenesis (Qi et al., 2021). Additionally, m6A mutations have a function in non-coding RNA regulation processes. METTL3, a m6A methyltransferase, enhances angiogenesis by boosting splicing of the precursor miR-143-3p, increasing miR-143-3p expression, and inhibiting VASH1 expression (Wang H et al., 2019). These findings imply that the RNA modification pathways involved in m6A in ocular neovascularization will be gradually elucidated in the near future.

## 4 Concluding remarks

The mechanisms of epigenetic changes such as DNA methylation, histone modifications, and non-coding RNA regulation in ocular neovascularization remain unknown. More in-depth study, however, will eventually reveal complex epigenetic regulatory processes in ocular neovascularization. The present regulatory network linking DNA methylation and RNA modifications of histones, noncoding RNAs, and m6A in ocular neovascularization, including epigenetics, is the subject of this study.
